# Multidrug-Resistant CTX-M and CMY-2 Producing *Escherichia coli* Isolated from Healthy Household Dogs from the Great Metropolitan Area, Costa Rica

**DOI:** 10.1089/mdr.2020.0146

**Published:** 2020-10-28

**Authors:** María José Rodríguez-González, María Antonieta Jiménez-Pearson, Francisco Duarte, Tomás Poklepovich, Josefina Campos, Luis Nazario Araya-Sánchez, Manuel Chirino-Trejo, Elías Barquero-Calvo

**Affiliations:** ^1^ Programa de Investigación en Enfermedades Tropicales, Escuela de Medicina Veterinaria, UNA, Heredia, Costa Rica.; ^2^ Instituto Costarricense de Investigación y Enseñanza en Nutrición y Salud, San José, Costa Rica.; ^3^ Plataforma Genómica y Bioinformática-Genomic and Bioinformatics Platform INEI-ANLIS “Dr. Carlos G. Malbrán, Buenos Aires, Argentina.; ^4^ Escuela de Medicina Veterinaria, Universidad Nacional, Heredia, Costa Rica.; ^5^ Western College of Veterinary Medicine, University of Saskatchewan, Saskatoon, Canada.

**Keywords:** *E. coli*, ESBL, CTX-M, AmpC, CMY-2, quinolones, dogs, antibiotics

## Abstract

***Objective:*** This study aimed to determine the prevalence of fecal carriage of antibiotic-resistant *Escherichia coli* of healthy household dogs with an emphasis on extended-spectrum β-lactamases (ESBL), AmpC-type β-lactamases and resistance to quinolones.

***Materials and Methods****:* Rectal swabs were collected from 74 dogs without any clinical evidence of gastrointestinal disease. Samples were cultured on MacConkey agar plates and MacConkey supplemented with 2 μg/mL cefotaxime or 5 μg/mL ciprofloxacin. Isolates were identified with Vitek 2 Compact and susceptibility testing performed by Kirby Bauer disk diffusion method. Minimal inhibitory concentration (MIC) was done on isolates resistant to cefotaxime, ciprofloxacin, and nalidixic acid. PCR amplification was performed to detect CTX-M and CMY-2. Isolates positive for CTX-M and/or CMY-2 were selected for whole-genome sequencing.

***Results:*** Multiresistance was detected in 56% of the isolates. A high percentage of resistance was detected for cefazolin (63%), ampicillin (54%), streptomycin (49%), nalidixic acid (42%) and tetracycline (38%). The MIC_50_ and MIC_90_ for isolates resistant to cefotaxime (24%) was determined as 16 and >250 μg/mL, respectively; for ciprofloxacin (18%), 125 and 250 μg/mL, respectively. ESBL (CTX-M type) and AmpC (CMY-2 type) were detected in 6 (7.1%) and 14 (19%) of the isolates, respectively. Whole-genome sequence analysis showed high genetic diversity in most of the isolates and a large variety of resistance mechanisms, including mobile genetic elements.

***Conclusion:*** The frequency of multidrug-resistant *E. coli* is worrying, mainly because of the presence of many isolates producing ESBL and AmpC β-lactamases. Based on the “One Health” concept, considering the relationships between animals, humans, and the environment, these data support the notion that companion animals are important reservoirs of multidrug-resistant bacteria.

## Introduction

The increasing incidence of multidrug-resistant commensal and pathogenic bacteria is a global concern. Infections caused by antibiotic-resistant bacteria increase mortality and morbidity in humans and animals. This emerging problem is increasing in part due to the indiscriminate use of antibiotics in humans and veterinary medicine. As hypothesized by O'Neill,^1^ 10 million people will die every year due to antimicrobial resistance by 2050 unless a global response to the problem is implemented.

The use of identical or similar antibiotics in animals and humans can have a negative impact on the selection of bacteria resistant to antibiotics intended for human use. Currently, many of the antibiotics used to treat human pathogens are also used in pets, agricultural and livestock industry, crop fertilization, and animal breeding.^2^ Despite the importance of β-lactams and quinolones in human medicine,^3^ these antibiotics are also one of the main groups of antibiotics used to treat dogs.^4,5^ This is mainly due to the importance of these molecules to treat serious animal diseases, but also to the lack of availability of alternative antimicrobial agents.^6^ This is also related to the lack of development of new antimicrobials that could be separately used for humans and animals.

The main mechanism of resistance to β-lactams is the production of β-lactamases. These enzymes hydrolyze the amide bond of the β-lactam nucleus, producing acid derivatives without a bactericidal effect.^7–9^ Extended-spectrum β-lactamases (ESBLs) and AmpC type enzymes are frequently isolated from extended-spectrum cephalosporin (ESC)-resistant Gram-negative bacteria. ESBL enzymes provide multiple resistance to β-lactams, including penicillins, monobactams, and many cephalosporins from the first to the fourth generation, except cefoxitin. They are strongly inhibited by clavulanic acid.^8^ Among the members of the ESBL β-lactamase family, the CTX-M β-lactamases are the most widespread enzymes among antibiotic-resistant bacteria.^10^ CTX-M family constitutes a complex and nonhomogeneous group of enzymes that are divided into five main groups based on similarities in amino acid sequence.^11^ AmpC-β-lactamases have a broader resistance against cephalosporins, including cephamycins (cefoxitin and cefotetan) and are not inhibited by clavulanic acid but are inhibited by cloxacillin.^8^

Quinolones are among the most commonly used antimicrobials in both human and veterinary clinical medicine.^5^ The mechanisms of resistance to quinolones include mutations altering drug targets, reduced drug accumulation, and plasmids-mediated genes that protect targets from the effects of quinolones.^12^

Owing to the close relationship with humans, dogs may be at risk of acquiring resistant strains from humans, including ESBL and AmpC-producing and/or quinolone-resistant *Escherichia coli*, although this is expected to be a two-way route.^13^ Resistance to ESCs and co-resistance to quinolones limits the treatment options for infections with ESBL/AmpC-producing bacteria.^14^ Furthermore, the use of ESCs and quinolones in dogs is known to select ESBL/pAmpC producers in the fecal microbiota.^15,16^

There are few studies in Latin America focusing on the prevalence of bacterial resistance in companion animals.^17^ Considering that companion animals can be a potential reservoir of antibiotic-resistant bacteria or resistance determinants, this study aimed to determine the prevalence of fecal carriage of antibiotic-resistant *E. coli* of healthy household dogs with an emphasis on ESBL, AmpC-type β-lactamases, and resistance to quinolones.

## Materials and Methods

### Sample collection

Rectal swabs were collected from 74 dogs from January to April 2015 in the urban region of the Greater Metropolitan Area, Costa Rica. Dogs were selected at random regardless of their breed, size, or age. Samples were taken at veterinary clinics or households from healthy dogs (without any clinical evidence of gastrointestinal disease) that had not been treated with antibiotics for at least 3 months before sampling. Only one dog was sampled per household. Samples were collected directly from the rectum using sterile commercial swabs with Stuart transport medium (Oxoid, Hampshire, United Kingdom). Swabs were kept under refrigeration and transported to the Laboratorio de Bacteriología, Escuela de Medicina Veterinaria, Universidad Nacional, within 24–72 hours after sampling.

### Isolation and identification of *E. coli*

Samples were plated directly on standard MacConkey (Oxoid) agar and MacConkey supplemented with 2 μg/mL cefotaxime (Sigma-Aldrich, MO) or with 5 μg/mL ciprofloxacin (Sigma-Aldrich) and incubated for 24 hours at 37°C. Colonies growing on MacConkey with cefotaxime or ciprofloxacin were selected over colonies growing on MacConkey without antibiotics. Up to two isolates per sample were analyzed if *E. coli* colonies were obtained in both media with antibiotics. Isolates with identical susceptibility profiles from the same sample were eliminated from the analysis. Typical lactose positive *E. coli* colonies were identified with Vitek 2 Compact (BioMèrieux, Marcy-l'Étoile, France) using the gram-negative card.

### Antibiotic susceptibility testing and minimal inhibitory concentration determination

Susceptibility testing was performed by Kirby Bauer disk diffusion method using 22 antibiotics: amikacin, ampicillin, cefpodoxime, ceftazidime, ceftriaxone, cefuroxime, ciprofloxacin, ertapenem, imipenem, nalidixic acid, nitrofurantoin, tetracycline, trimethoprim/sulfamethoxazole (Becton Dickinson, NJ), ampicillin-sulbactam, aztreonam, cefazoline, cefepime, cefotaxime, ceftiofur, chloramphenicol, kanamycin, and streptomycin (Oxoid), according to the Clinical and Laboratory Standards Institute, CLSI.^18^ ESCs and ciprofloxacin-resistant isolates were selected and cryopreserved for further analysis. Multiresistance was defined as the resistance to three or more structural classes of antibiotics^19^ and M100 from CLSI (30th edition, 2020) was used for interpretation and antimicrobial class classification (Glossary I).^20^ Isolates showing an intermediate resistant pattern were included in the resistant group for the analysis of antibiotic susceptibility but excluded from the minimal inhibitory concentration (MIC) assays. MICs were performed manually using the microdilution method on isolates that showed resistance to cefotaxime, ciprofloxacin, and nalidixic acid as recommended by CLSI.^21^ Concentrations from 0.125 (μg/mL) to 256 (μg/mL) were evaluated for each antibiotic.

### Detection of ESBL (*bla*_CTX-M_) and AmpC (*bla*_CMY-2_) isolates

ESBL detection was performed using the double-disk synergy test with cefotaxime (30 μg) and ceftazidime (30 μg) disks with and without clavulanate (10 μg).^20^ CTX-M detection was performed using the methodology previously described.^22^ CTX-M PCR amplification of *bla*_CTX-M_ alleles was carried out with primers CTX-MU1 (5′-ATG TGC AGY ACC AGT AAR GT-3′) and CTX-MU2 (5′-TGG GTR AAR TAR GTS ACC AGA-3′). These PCR primers allow the amplification of a 593 bp fragment present in different types of CTX-M, including *bla*_CTX-M-1_ to *bla*_CTX-M-30_.

AmpC detection was performed using the AmpC Confirm Kit (ROSCO Diagnostica, Taastrup) based on disk diffusion tablets. CMY-2 detection was performed using the methodology previously described.^23^ PCR was performed with the primers CMY-2F (5′-TGG CCA GAA CTG ACA GGC AAA-3′) and CMY-2R (5′-TTT CTC CTG AAC GTG GCT GGC-3′). These PCR primers allow the amplification of a 462 bp fragment, including *bla*_CMY-2_. The strains *Shigella flexneri* OPS 187 and *Proteus mirabilis* OPS 146 were used as positive *bla*_CTX-M_ and *bla*_CMY-2_ controls, respectively. *E. coli* ATCC 25922 was used as a negative control for both PCRs. QIAxcel Advanced automated system was used to visualize all PCR fragments.

### Whole-genome sequence

Whole-genome sequencing was performed at MicrobesNG on Illumina HiSeq platforms using a 250 bp paired-end protocol. Reads quality was checked with FastQC and genome assembly with Unicycler,^24^ with assembly quality check using Quast^25^ and annotation with Prokka.^26^ For evaluating the clonal relationship, a core-genome alignment from the assembled genomes was created using Roary^27^ and SNPsites^28^ to determine SNPs in the core-genome alignment. The maximum likelihood tree was built using RAxML.^29^ Serotype was determined using SRST2^30^ against EcOH database from Holt Lab. ARIBA^31^ was used for multilocus sequence typing (MLST) analysis according to the Achtman scheme and with ResFinder^32^ reference database to identify resistance genes. Point mutation resistance was investigated using AMRFinderPlus^33^ and plasmid detection and typing using PlasmidFinder.^34^

### Bioethical considerations

This study was carried out with the approval of the Ethics Committee of the School of Veterinary Medicine, Universidad Nacional (FCSA-EMV-CBAB-001-2015) and by the corresponding law, Ley de Bienestar de los Animales of Costa Rica (law 9458 on animal welfare). Informed consent was obtained from each dog owner.

## Results

### Multidrug-resistant *E. coli*

**FIG. 1.**
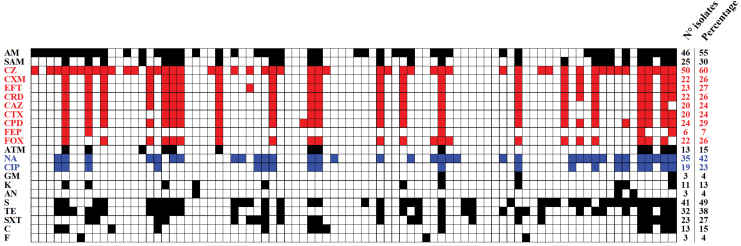
Antibiotic resistance patterns. *Full colored squares* indicate resistance and *white squares* represent susceptibility. *Red squares* and *blue squares* belong to the cephalosporin and quinolone classes, respectively. AM, Ampicillin; SAM, ampicillin/sulbactam; CZ, cefazolin; CXM, cefuroxime; EFT, ceftiofur; ceftriaxone, CRD; ceftazidime, CAZ; cefotaxime, CTX; cefpodoxime, CPD; cefepime, FEP; FOX, cefoxitin; ATM, aztreonam; NA, nalidixic acid; CIP, ciprofloxacin; GM, gentamicin; K, kanamycin; AN, amikacin; S, streptomycin; TE, tetracyclin; SXT, trimetroprim/sulfamethoxazole; C, chloramphenicol; F, nitrofurantoin. Color images are available online.

**FIG. 2.**
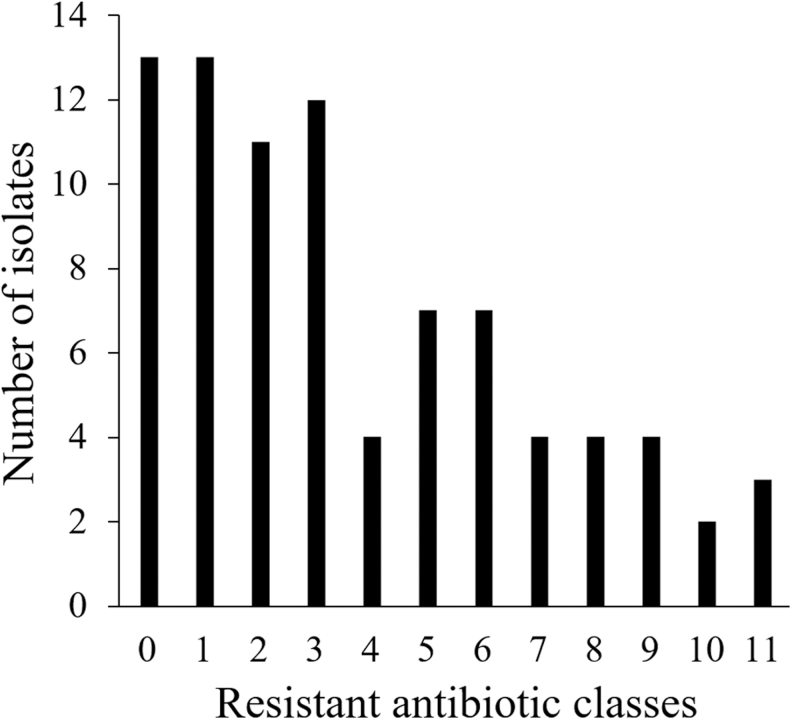
Resistant antibiotic classes per number of isolates.

Eighty-four *E. coli* isolates were recovered and subjected to antibiotic susceptibility testing. Twenty-four percent and 18% of the isolates were resistant to cefotaxime and ciprofloxacin, respectively. Besides, >50% of the isolates presented resistance to cefazolin and ampicillin. Likewise, a high percentage of resistance was found for streptomycin (49%), nalidixic acid (42%), and tetracycline (38%). In total, 23–30% of the isolates presented resistance against ampicillin-sulbactam, second- and third-generation cephalosporins, trimethoprim/sulfamethoxazole, and ciprofloxacin. Combined resistance cefotaxime and ciprofloxacin were observed in 18% of the isolates. Other antibiotics of infrequent use in veterinary medicine showed percentages of resistance of 15% or less; chloramphenicol and aztreonam (15%), aminoglycosides (4–13%), cefepime (7%), and nitrofurantoin (4%) (Fig. 1). None of the isolates were resistant to ertapenem or imipenem (not shown). As observed in Fig. 1, resistance profiles among isolates were very heterogeneous. No predominant phenotypic resistant antibiotic profile was observed. Multidrug resistance was detected in 56% of the isolates, including 17 isolates resistant to 7 or more antibiotics classes. Thirteen isolates (15%) were pansusceptible (Fig. 2).

The MIC analyses showed different patterns for the anbiotics evaluated. The MIC_50_ and MIC_90_ calculations indicated the following results: ciprofloxacin (MIC_50_ 125 μg/mL, MIC_90_ 256 μg/mL), nalidixic acid (MIC_50_ >256 μg/mL, MIC_90_ >256 μg/mL), and cefotaxime (MIC_50_ 16 μg/mL, MIC_90_ >256 μg/mL).

### ESBL (*bla*_CTX-M_) and AmpC (*bla*_CMY-2_) detection

Six (7.1%) ESBL-producing isolates were detected using the double-disk synergy method. Detection of *bla*_CTX-M_ genes was confirmed in these isolates (not shown). Likewise, in all 14 (16.7%) isolates positively identified by the AmpC Confirm Kit, the gene encoding for the plasmid-mediated AmpC β-lactamase CMY-2 was detected.

### Whole-genome sequence analysis

**FIG. 3.**
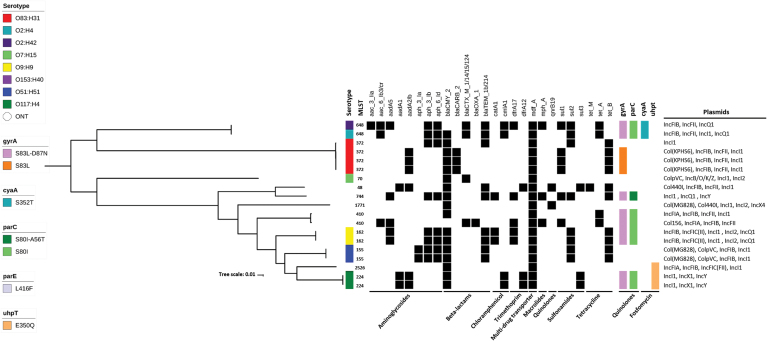
Reconstructed phylogeny based on the core genome of nineteen *Escherichia coli* isolates. The MLST genotype and the serotype of each isolate (color-labeled) are indicated in its respective branch. The presence and absence of selected antimicrobial resistance genes are shown, *black* indicating presence. Point mutations conferring antimicrobial resistance (color-labeled) and plasmids are also indicated. Antimicrobial classes are shown at the *bottom* of the figure. MLST, multilocus sequence typing. Color images are available online.

Whole-genome sequencing was performed in 19 CTX-M and CMY-2 producing isolates. The phylogeny analysis showed high genetic diversity among isolates. Few isolates clustered together. Clustering correlated, most of the time, with the serotype, the MLST genotype and the mechanisms conferring resistance (genes and point mutations) (Fig. 3). The presence of CTX-M (*bla*_CTX-M-1_, *bla*_CTX-M-14_, *bla*_CTX-M-15_, and *bla*_CTX-M-124_) and CMY-2 mechanisms, previously detected by PCR (not shown), was confirmed. Other β-lactamase genes encoding for broad-spectrum enzymes such us *bla*_CARB-2_, *bla*_OXA-1_, and *bla*_TEM-1_ were also detected. Even if the isolate selection for sequencing was based on ESBL and AmpC producing isolates, many other mechanisms were also detected, including plasmid-mediated quinolone resistance (*qnr*) and quinolone resistance mutations in the *gyrA* and *parC* genes (Fig. 3). The most common plasmid types were IncI1 (89.5%) and IncFIB (68.4%). Furthermore, a range of plasmids previously associated with multidrug-resistant bacteria: IncFII (36.8%), IncQ1 (26.3%), Incl2 (21.1%), IncFIA, IncY, IncFIC(FII) and ColpVC Col(KPHS6) (15.8%), InX1 (10.5%) and IncB/O/K/Z, Inx4, Col156 (5.3%) (Fig. 3).

## Discussion

The antibiotic resistance profiles obtained in this study (Fig. 1) are similar to those previously reported in dogs in other countries with some differences. A study conducted in Colombia also reported a high percentage of resistance in tetracyclines but lower percentages of resistance to aminopenicillins and aminoglycosides, such as streptomycin.^35^ In a study in Portugal, resistance against ampicillin was also reported as one of the most prevalent but reported a higher percentage of resistance against cefotaxime, trimethoprim/sulfamethoxazole, cefuroxime, tetracycline, streptomycin, and gentamicin.^36^ Resistance to fourth-generation cephalosporins, nitrofurantoin, and aminoglycosides was very infrequent in many studies. This is probably due to the restricted use of these drugs in animals and humans.^36–38^

In comparison with other studies, the percentage of multidrug resistance in this study was very high (56%). Schmidt *et al.* reported a prevalence of 30% of multidrug-resistant isolates in Labrador dogs in the United Kingdom,^37^ whereas Leite-Martins *et al.* reported <13% of multidrug-resistant *E. coli* isolates.^36^ In contrast, a study conducted in Poland reported an alarming 66.8% of multiresistance.^38^

In this study, we did not observe a predominant phenotypic antibiotic-resistant profile (Fig. 1). As proposed by Albrechtova *et al.*, this is likely due to the dissemination of several bacterial strains and not the success of a single multidrug-resistant clonal bacterium.^39^ The number of antibiotic classes was also very variable, from pansusceptible isolates (*n* = 13) to isolates with resistance to 10 (*n* = 2) or 11 (*n* = 3) different antibiotic classes (Fig. 2).

According to the MIC analyses, many of the quinolone-resistant isolates were highly resistant. The breakpoint for ciprofloxacin in the CLSI guide at the time that this study was carried out (2015) was ≥4 μg/mL. Accordingly, this was the breakpoint selected for isolation and interpretation of the isolates. This is a limitation of this study since the current breakpoint is lower (≥1 μg/mL). Consequently, that resistance for ciprofloxacin and nalidixic acid could be underestimated. Whole-genome sequencing confirmed the presence of point mutations in the *gyrA* and *parC* genes in many isolates and the presence of plasmid-mediated quinolone resistance (Fig. 3).

The high co-resistance to cefotaxime and ciprofloxacin detected in this study is a concern. Other studies have also reported co-resistance of quinolones and cephalosporins in clinical isolates of *E. coli*^40^; in some cases, resistance genes were present in transferable plasmids, which increases the risk of dissemination of such genes.^41^ The presence of carbapenemases was discarded since no isolates showed a resistant phenotype against this antibiotic class. Owing to the importance of carbapenems in the treatment of Gram-positive and Gram-negative bacteria and the emergence and rapid spread in all continents of carbapenem resistance,^42^ the absence of carbapenemases in this study is of particular relevance.

The isolation of CTX-M ESBL-producing *E. coli* is consistent with previous reports in dogs.^43^ This enzyme was detected for the first time in 1980 in the fecal samples of a dog in Japan, and since 2000 there has been a global spread.^44^ The most common subtype reported so far is the CTX-M-15 type enzyme^37^ and is currently detected in this study. In Costa Rica, CTX-M has been confirmed in *E. coli* isolates from clinical cases in humans, as well as in other *Enterobacteriaceae* such as *Klebsiella pneumoniae*, all through laboratory-based surveillance carried out at the National Reference Center of Bacteriology at INCIENSA.^45^

Similarly, CMY-2 AmpC-producing *E. coli* has been previously reported in dogs in many countries, including household dogs.^46^ The percentage of detection of CMY-2 is variable among studies, ranging from <1% detection up to 20%.^47^ In this study, we detected 19% of CMY-2 AmpC-producing *E. coli.* This is remarkable since CMY-2-type plasmid-mediated AmpC β-lactamase has been recently reported emerging in Costa Rica in *Shigella sonnei* and *Salmonella* spp.^46^ and was also confirmed in clinical isolates of *E. coli* from humans by INCIENSA through laboratory-based surveillance.^45^

Among the MLST genotypes reported in this study, some of them have been isolated in both humans and animals; in particular, ST372, ST410, and ST224 have been reported previously in humans and pets.^48,49^ This shows that these, and probably many other *E. coli* genotypes carrying antimicrobial resistance genes, can be shared between animals and humans.

Owing to the widespread use of beta-lactams and quinolones for treating infections in dogs, the resistance mechanisms detected in this study are highly relevant and, through direct contact, have the potential risk of transmission of bacteria or gene determinants to humans or vice versa. This is especially significant in mechanisms present in genetic mobile elements in *E. coli* such as ESBL and AmpC beta-lactamases found in plasmids.

Based on the “One Health” concept, considering the relationships between animals, humans, and the environment, these data support the notion that companion animals are important reservoirs of multidrug-resistant bacteria. To our knowledge, this is the first report to show fecal carriage of multidrug-resistant ESBL and AmpC-producing *E. coli* isolated from household dogs in Central America. Further studies with human and animal isolates are required in this geographical area to better understand the epidemiology and relevance of these strains in public and animal health.
